# Ponicidin Promotes Hepatocellular Carcinoma Mitochondrial Apoptosis by Stabilizing Keap1‐PGAM5 Complex

**DOI:** 10.1002/advs.202406080

**Published:** 2024-08-08

**Authors:** Bixin Zhao, Zuhui Liang, Lisheng Zhang, Lin Jiang, Yuanhang Xu, Ying Zhang, Rong Zhang, Caiyan Wang, Zhongqiu Liu

**Affiliations:** ^1^ State Key Laboratory of Traditional Chinese Medicine Syndrome International Institute for Translational Chinese Medicine Guangzhou University of Chinese Medicine Guangzhou 510006 China; ^2^ Research Center of Integrative Medicine School of Basic Medical Science Guangzhou University of Chinese Medicine Guangzhou 510006 China

**Keywords:** apoptosis, Hepatocellular carcinoma, Keap1, mitochondrial function, PGAM5, ponicidin

## Abstract

Ponicidin is a diterpenoid with demonstrated antitumor activity in clinical trials. However, the specific function and mechanism of action against hepatocellular carcinoma (HCC) remain unknown. In this study, it is found that ponicidin significantly inhibited the proliferation and migration of HCC cells. It is shown that ponicidin targets Keap1 and promotes the formation of the Keap1‐PGAM5 complex, leading to the ubiquitination of PGAM5, using biotin‐labeled ponicidin for target fishing and the HuProt^TM^ Human Proteome Microarray V4.0. Ponicidin is found to activate the cysteine‐dependent mitochondrial pathway via PGAM5, resulting in mitochondrial damage and ROS production, thereby promoting mitochondrial apoptosis in HepG2 cells. The first in vitro cocrystal structure of the PGAM5 IE 12‐mer peptide and the Keap1 Kelch domain is obtained. Using molecular dynamics simulations to confirm the binding of ponicidin to the Keap1‐PGAM5 complex. Based on the depth‐based dynamic simulation, it is found that ponicidin can induce the tightening of the Keap1‐PGAM5 interaction pocket, thereby stabilizing the formation of the protein complex. Finally, it is observed that ponicidin effectively inhibited tumor growth and promoted tumor cell apoptosis in a BALB/c nude mouse xenograft tumor model. The results provide insight into the anti‐HCC properties of ponicidin based on a mechanism involving the Keap1‐PGAM5 complex.

## Graphical abstract

Targeted therapy for hepatocellular carcinoma (HCC) is a new therapeutic strategy. Structural biology and molecular dynamics simulations have demonstrated that ponicidin can target Keap1, stabilize the formation of Keap1‐PGAM5 complex, and thus induce mitochondrial apoptosis. Ponicidin is a potential small molecule for the targeted treatment of HCC.

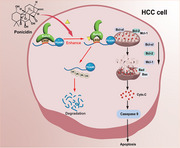

## 1 Introduction

Hepatocellular carcinoma (HCC) is a primary hepatic malignancy associated with chronic liver disease and cirrhosis.^[^ ^1^ ^]^ The incidence of HCC has risen sharply in recent years, becoming the fifth most common malignancy worldwide and a leading cause of cancer‐related mortality.^[^ ^2^ ^]^ The correlation between incidence and mortality (830000 deaths per year) serves to illustrate the unfavorable prognosis associated with this disease.^[^ ^3^ ^]^ Unfortunately, HCC is often difficult to detect in its early stages, with most cases being diagnosed at intermediate or late stages. Currently, immunotherapy and chemotherapy are the best available treatment options for patients.^[^ ^4^ ^]^ Furthermore, the majority of patients diagnosed with HCC experience a recurrence following surgical resection or ablation, or are initially diagnosed at an advanced stage.^[^ ^5^ ^]^ Therefore, there is an urgent need for more effective treatment options for patients with HCC, and molecularly targeted therapy is a promising approach that may provide better outcomes with reduced systemic toxicity and fewer side effects than current therapies.^[^ ^6^ ^]^

PGAM5 is a mitochondrial protein involved in various cellular processes such as cell death, metabolism, and stress response.^[^ ^7^, ^8^, ^9^, ^10^, ^11^ ^]^ Elevated levels of PGAM5 are strongly associated with HCC, melanoma, non‐small cell lung cancer and gastric cancer,^[^ ^12^, ^13^, ^14^, ^15^ ^]^ but the precise role of PGAM5 in HCC is unclear. PGAM5 consists mainly of a transmembrane structural domain, a linker domain containing a regulatory multimerization motif, an NxESGE motif and a PGAM domain.^[^ ^16^ ^]^ The NxESGE motif of PGAM5 interacts with the Kelch domain of Keap1 and its C‐terminus binds to Bcl‐xL. Keap1‐dependent ubiquitination leads to proteasome‐dependent degradation of PGAM5 and Bcl‐xL, thereby inducing cell apoptosis.^[^ ^17^ ^]^ The current study demonstrates that Keap1‐mediated ubiquitination degradation of PGAM5 is active in human colorectal cancer (CRC).^[^ ^18^ ^]^ Furthermore, the Keap1‐PGAM5 signaling pathway has been investigated in human ovarian cancer,^[^ ^19^ ^]^ breast cancer,^[^ ^20^ ^]^ heart failure,^[^ ^21^ ^]^ and myocardial ischemia‐reperfusion injury.^[^ ^22^ ^]^ Targeting the novel signaling pathway mediated by the Keap1‐PGAM5 complex may represent a novel therapeutic strategy to improve the survival outcomes of HCC patients.

Ponicidin is a diterpenoid compound extracted from *Rabdosia rubescens*. Ponicidin has demonstrated various beneficial immunological activities, including antiviral, immunomodulatory, anti‐inflammatory, and antitumor effects.^[^ ^23^ ^]^ Previous work has also shown that ponicidin can inhibit the growth of pancreatic cancer cells by affecting cell metabolism and ferroptosis‐mediated cell death.^[^ ^24^ ^]^ Regarding cancer, ponicidin has been shown to induce apoptosis in gastric cancer cells by affecting specific signaling pathways such as the JAK2 and STAT3 signaling pathways.^[^ ^25^ ^]^ Another study showed that ponicidin activated the p38 pathway and its downstream targets such as caspase 3 and Bax to induce apoptosis of colon cancer cells.^[^ ^26^ ^]^ Despite ponicidin's efficacy against multiple malignancies, its precise function and mechanism of action in relation to HCC remain unknown.

The objective of this study was to investigate the effect of ponicidin on PGAM5 function in HCC development. Our data suggest that ponicidin facilitates the binding of Keap1 and PGAM5. We resolved the crystal structure of the Kelch structural domain of Keap1 with the PGAM5 12‐mer peptide, obtained the binding mode and complete structure of the Keap1‐PGAM5 complex from experimental and computational data, and confirmed through molecular dynamics simulations that ponicidin binds to it. It was found that ponicidin can impact the structural changes of the Kelch domain where Keap1 interacts with PGAM5, promoting the binding stability of Keap1 and PGAM5, and subsequently affecting the ubiquitination and degradation of PGAM5. Furthermore, ponicidin activates the caspase – dependent mitochondrial pathway via PGAM5, leading to mitochondrial damage and ROS production‐related pathways thereby promoting mitochondrial apoptosis in HepG2 cells. In a BALB/c nude mice xenograft tumor model, ponicidin effectively inhibited tumor growth and promoted tumor cell apoptosis. These findings suggest that ponicidin has potential as a treatment for HCC and warrants further investigation.

## 2 Results

### 2.1 Ponicidin Inhibited the Proliferation and Migration of HCC Cells

**Figure 1:**
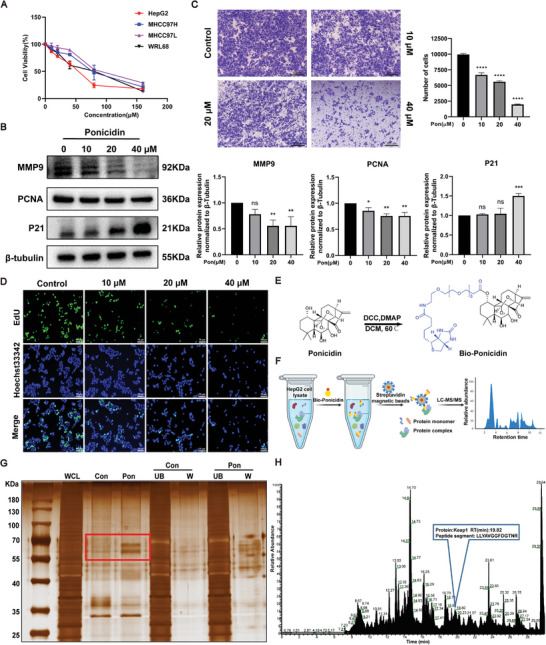
Effect of ponicidin on the proliferation and migration of HepG2 cells. A) The viability of cells was quantified by the MTT assay following exposure to varying concentrations of ponicidin for a period of 24 h. B) Representative western blotting images of the expression levels of PCNA, P21, and MMP9. C) Representative images of HepG2 cells after transwell assay. D) EdU staining was used to assess the effect of ponicidin on the proliferation of HepG2 cells. E) Biochemical reaction for obtaining biotin‐labeled ponicidin was diagrammed. F) The schematic shows the steps for identifying ponicidin‐binding proteins in HepG2 cell lysates using target fishing techniques. G) Proteins pulled down by biotin‐labeled probe for ponicidin and control probe were analyzed by silver staining. WCL: whole cell lysates; Con: proteins bound to magnetic beads; Pon: ponicidin‐binding proteins; UB: the unbound proteins; W: the washing proteins. H) The pulled‐down proteins were identified by mass spectrometry analysis. These experiments were repeated three times (n = 3), with ^*^P < 0.05, ^**^P < 0.01, ^***^P < 0.001, ^****^P < 0.0001 via one‐way analysis of variance (ANOVA).

To investigate the effect of ponicidin on cell viability, we performed an MTT assay. The cytotoxicity experiments revealed that the inhibitory effect of ponicidin on the proliferation of HCC cells and WRL68 increased with increasing concentrations of ponicidin treatment. The IC_50_ of ponicidin in HepG2 cells was the lowest among the three liver cancer cell lines tested, with values of 77.5 µM for MHCC97H, 94.1 µM for MHCC97L, and 48.2 µM for HepG2 (**Figure** **1**A). Protein analysis of HepG2 cells treated with ponicidin revealed a dose‐dependent decrease in the expression of the proliferative marker PCNA and an increase in the expression of the cell cycle repressor protein P21. Furthermore, the expression of the migration marker MMP9 gradually decreased with increasing concentrations of ponicidin treatment (Figure 1B). Transwell experiments demonstrated that the number of cells passing through the chambers decreased with increasing concentrations of ponicidin treatment. This indicates that ponicidin inhibited the migration of HepG2 cells in a dose‐dependent manner (Figure 1C). EdU staining showed that the proportion of EdU‐positive cells decreased in a concentration‐dependent manner in the presence of ponicidin, indicating that ponicidin inhibited the proliferative activity of HepG2 cells (Figure 1D; Figure S1, Supporting Information). The data indicate that ponicidin can effectively inhibit the proliferation and migration of HepG2 cells in a dose‐dependent manner. To confirm the target of ponicidin on HepG2 cells, we synthesized biotin‐labeled ponicidin (Bio‐Ponicidin) (Figure 1E). HepG2 cell lysates were incubated with biotin‐labeled ponicidin. Proteins that could bind ponicidin were subsequently pulled down with streptavidin magnetic beads, and silver staining analysis showed the presence of a differential band near 70 KDa (Figure 1F,G). We then identified the ponicidin‐bound proteins using mass spectrometry analysis, which included Keap1 protein (69 KDa) (Figure 1H). The results of this analysis suggested that ponicidin may target Keap1.

### 2.2 PGAM5 is Upregulated in HCC Tissue Samples

**Figure 2:**
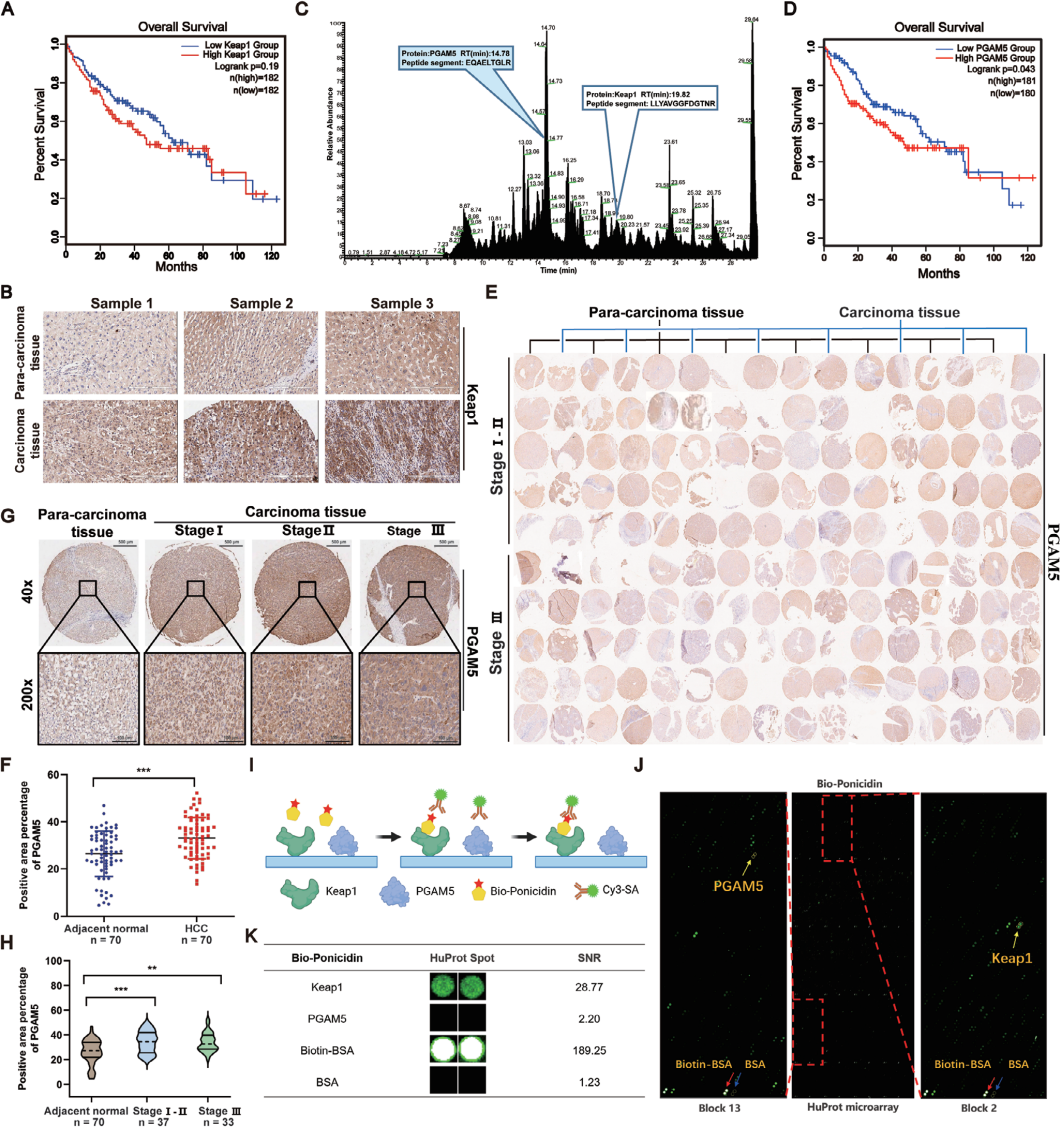
Expression and prognostic significance of PGAM5 in HCC samples from patients. A) Analysis of the association of high or low Keap1 expression with overall survival in HCC. B) Representative images of Keap1 immunohistochemistry on the Tissue microarray (TMA) are shown (original magnification: 40x). C) The pulled‐down proteins were identified by mass spectrometry analysis. D) Analysis of the association of high or low PGAM5 expression with overall survival in HCC. E) TMA of HCC. F) Comparison of PGAM5 expression in adjacent normal tissues (n = 70) and HCC tissues (n = 70). G) Representative images of PGAM5 immunohistochemistry on the TMA are shown (original magnification: upper: 40x; lower: 200x). H) Expression comparison of PGAM5 in the adjacent normal tissues (n = 70), Stage I‐II HCC (n = 37) and Stage III HCC tissues (n = 33). I) The schematic illustrates the methodology for identifying ponicidin‐binding proteins using microarrays fabricated with recombinant human proteins. J) Human protein microarrays were probed with Bio‐ponicidin. The binding was then detected by means of a Cy3‐labeled streptavidin. Control experiments were conducted with free biotin (green pots indicating interacting proteins). The red arrow indicates the positive control (Biotin‐BSA), the blue arrow indicates the negative control (BSA), and the yellow arrow indicates the target protein. K) Representative Keap1 and PGAM5 signals from the protein arrays. The signal‐to‐noise ratios (SNR) are presented. ^*^P < 0.05, ^**^P < 0.01, ^***^P < 0.001, ^****^P < 0.0001 via one‐way analysis of variance (ANOVA).

To investigate the correlation between Keap1 expression levels and the survival time of HCC patients, we analyzed the TCGA database (http://gepia2.cancer‐pku.cn/#index). Our findings suggest that high Keap1 expression is associated with a lower survival rate of HCC patients in the first 60 months. However, the relationship between Keap1 expression and survival rate was not significant in the last 60 months (**Figure** **2**A). Previous studies have shown that the higher Keap1 expression is closely related to longer progression‐free survival in non‐small cell lung cancer.^[^ ^27^ ^]^ However, recent studies have shown that higher Keap1 protein expression levels are closely related to lower survival time in colorectal cancer.^[^ ^28^ ^]^ In this study, we used a HCC liver tissue microarray to detect the expression of Keap1 and found that Keap1 expression in HCC tissues was higher than in para‐carcinoma tissues (Figure 2B). Keap1 protein is a crucial regulatory protein that can regulate intracellular signaling pathways by interacting with other proteins. Based on this, it is suggested that Keap1 may impact HCC through its interactions with other proteins. After conducting a literature search and analyzing previous mass spectrometry data, it was discovered that PGAM5 is a significant protein that interacts with Keap1 and can be pulled down by biotin‐labeled ponicidin (Figure 2C). Subsequently, the TCGA database was used to analyze the relationship between PGAM5 expression levels and the survival time of HCC patients. High expression of PGAM5 was associated with a lower survival rate of HCC (*P* = 0.043). These results suggest that PGAM5 plays a significant role in the pathogenesis of HCC (Figure 2D). We selected tissue microarrays from 80 patients diagnosed with hepatocellular carcinoma. Of these patients, 64 were male and 16 were female, with ages ranging from 16 to 76 years old. All samples were primary liver tumors and not metastatic hepatocellular carcinoma. The pathological section area covered almost all parts of the liver, and the pathological grade ranged from I to III. Immunohistochemical analysis was performed to assess the expression of Keap1 and PGAM5 in both HCC and adjacent normal tissues (Figure 2E). Representative images showed higher PGAM5 expression in HCC tissues compared to adjacent normal tissues. Quantitative analysis revealed a significant upregulation of PGAM5 in HCC tissues compared to adjacent normal tissues (Figure 2F).The intensity of positive staining was found to be correlated with higher pathological grading (Figure 2G). Further analysis of HCC tissue samples by stage, revealed upregulation of PGAM5 in all stages compared to adjacent normal tissues (Figure 2H). These results indicated a potential role of PGAM5 in the pathogenesis of HCC. Furthermore, we employed biotin‐labeled ponicidin (Bio‐Ponicidin) to discern the binding of ponicidin to recombinant proteins prepared on the HuProt^TM^ Human Proteome Microarray (Figure 2I,J). Bio‐ponicidin was incubated and detected using Cy3‐streptavidin (Cy3‐SA), after which the signal‐to‐noise ratio was calculated, defined as the ratio of foreground values to background values. A total of 662 proteins (SNR ≥ 13) were identified that bind to ponicidin (Figure S2, Supporting Information). Notably, ponicidin demonstrated a high binding affinity for the Keap1 protein (SNR = 28.77), whereas there was almost no binding to the PGAM5 protein (SNR < 13) (Figure 2K). Furthermore, we demonstrated that Keap1 knockdown in HepG2 cells resulted in a significant reduction in MMP9 protein expression when treated with ponicidin. This was observed in the siNC group. Nevertheless, the knockdown of Keap1 on HepG2 cells followed by ponicidin treatment had no significant effect on MMP9 protein expression (Figure S3, Supporting Information). The results indicate that ponicidin may bind directly to Keap1 and affect PGAM5, thereby exerting pharmacological effects in HCC.

### 2.3 The Structural Basis of the Interaction between Keap1 and PGAM5

**Figure 3:**
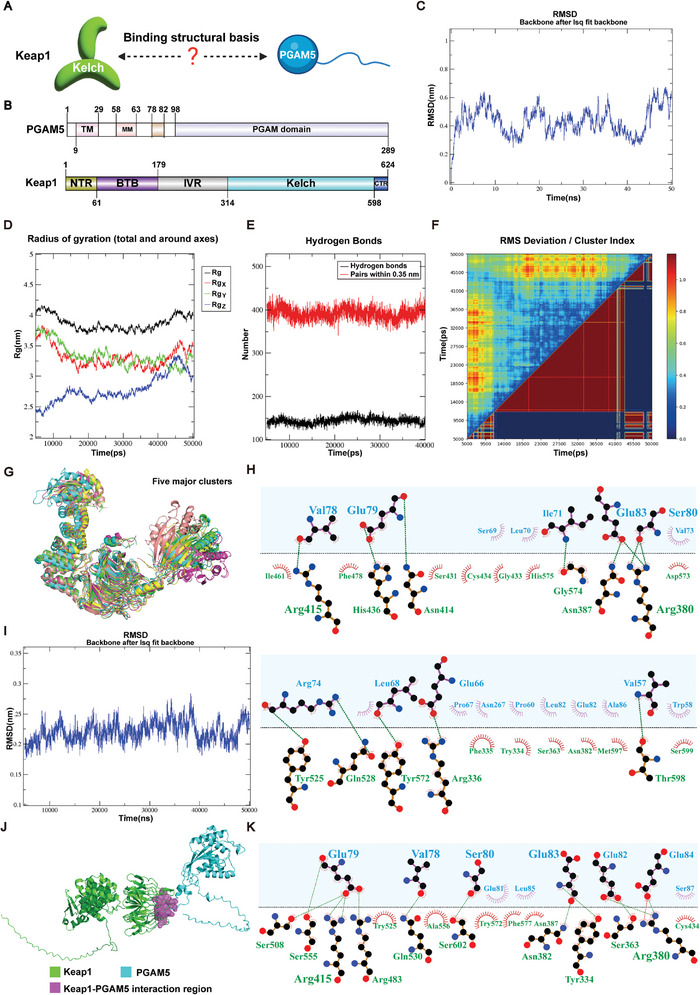
The structural basis of the interaction between Keap1 and PGAM5. A) The schematic diagram of the structural basis of the interaction between Keap1 and PGAM5. B) Domain organization of PGAM5 and Keap1. C) RMSD plot of the Keap1‐PGAM5 complex. D) Rg curves of Keap1‐PGAM5 complex. E) Hydrogen bonds and pairs of other intermolecular interactions in the interface between Keap1 and PGAM5. F) Clustering analysis of Keap1‐PGAM5 conformations. G) Representative structures from the five major clusters. Time is indicated by color: green (11570 ps), cyan (5000 ps), purple (5670 ps), yellow (39430 ps), and salmon (19970 ps). H) 2D visualization of the interactions at the interface between Keap1 and PGAM5. Amino acids from Keap1 and PGAM5 are colored in green and blue, respectively, and hydrogen bonds are depicted as green dashed line (PGAM5/Keap1). I) RMSD plot of the I71‐Y92 region in PGAM5. J) Schematic representation of the predicted regions of interaction between Keap1 and PGAM5 using AlphaFold3. K) 2D visualization of predicted Keap1 and PGAM5 interactions using AlphaFold3. Amino acids from Keap1 and PGAM5 are colored in green and blue, respectively, and hydrogen bonds are depicted as green dashed line (PGAM5/Keap1).

Studies have shown that Keap1 can bind to PGAM5. However, the structural basis of their binding is unclear (**Figure** **3**A). PGAM5, a member of the phosphoglycerate mutase superfamily, contains a PGAM domain, TM domain, MM domain, and an NxESGE motif that interacts with Keap1. Keap1, on the other hand, has NTR domain, BTB domain, IVR domain, Kelch domain and CTR domains, with the Kelch domain being responsible for binding to PGAM5 (Figure 3B). To observe the dynamic changes in the binding process of Keap1 and PGAM5, and to further study the structural basis of the binding of Keap1 to PGAM5, we conducted a molecular dynamics simulation. The root means square deviation (RMSD) curve of the Keap1‐PGAM5 complex indicates that the complex has high flexibility, however, the RMSD trajectory curve fluctuates around its mean, indicating that the structure has reached a stable state (Figure 3C). The structure of the Keap1‐PGAM5 complex remains generally stable (Figure 3D). The average number of hydrogen bond bindings is 14.3155, and the average number of other interaction pairs is 39.20747, indicating that many intermolecular interactions can be stably formed (Figure 3E). The trajectory was also clustered to investigate the movement patterns of the protein complex, obtaining five distinct clusters, and it was found that the structure of 16 520 ps is the most stable conformation. (Figure 3F,G). The interface binding at 16520 ps underwent further analysis, and many critical residues, revealing critical residues such as Arg380 and Asn414 on the Kelch domain of Keap1, and Glu79 and Val78 on PGAM5. Notably, the RMSD fluctuation in the PGAM5 (Ile71‐Tyr92) region was lower, indicating stability in the binding site sequence of PGAM5 (Ile71‐Tyr92) and Keap1 (Figure 3H,I). We employed the AlphaFold3 algorithm to predict the Keap1‐PGAM5 interaction.^[^ ^29^ ^]^ The resulting prediction yielded a pTM score of 0.53, indicating that the overall predicted folding of the complex is similar to the real structure. These findings align with those of molecular dynamics simulations, which revealed that the Val78, Glu79, Ser80, and Glu83 amino acids on PGAM5 primarily interact with the Kelch structural domain of Keap1 (Figure 3J,K).

### 2.4 Insight Analysis of the Crystal Overall Structure and Interaction in the Keap1‐PGAM5 Complex

**Figure 4:**
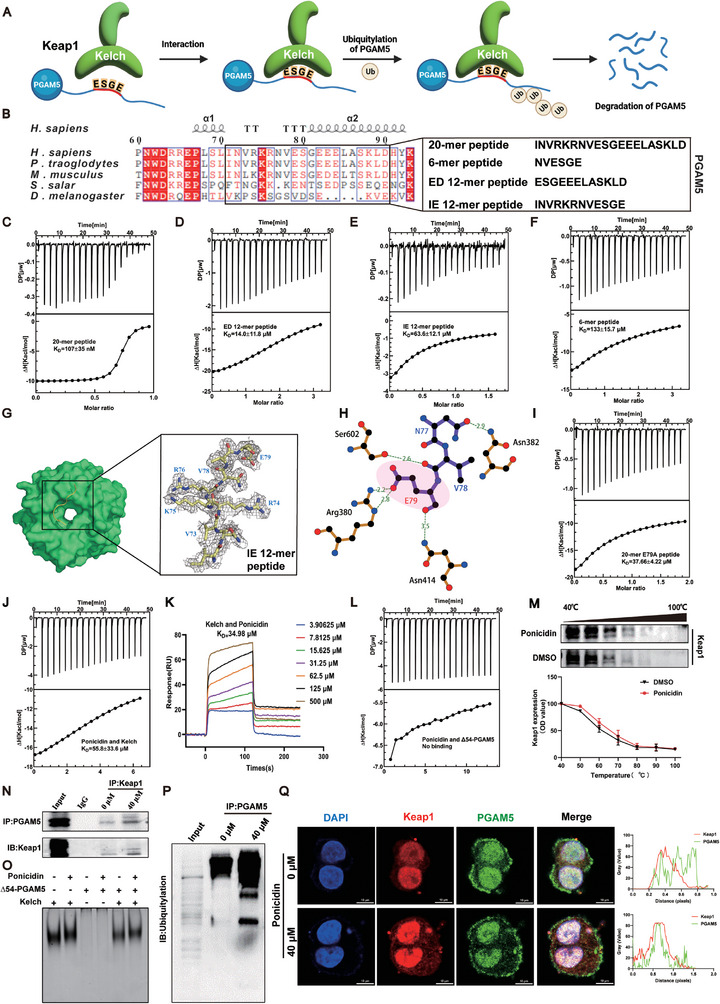
Ponicidin promoted the interaction between Keap1 and PGAM5 and promoted the ubiquitination of PGAM5. A) The binding diagram of Keap1 and PGAM5. B) Comparison of PGAM5 sequences from different species, including *Homo sapiens* (*H. sapiens*), *Pan troglodytes* (*P. troglodytes*), *Mus musculus* (*M. musculus*), *Salmo salar* (*S. salar*) and *Drosophila melanogaster* (*D. melanogaster*). Conserved residues are shown in red. Sequences of four peptides are shown below. C–F) ITC experiments of Kelch and four peptides, with affinity expressed as KD value. G) The overall structure of Kelch (green) with the IE 12‐mer peptide (yellow) on the left. On the right, the electron density map of the IE 12‐mer peptide is depicted in gray. The gray represents the electron density. H) A 3D plot of the interaction interface between the IE 12‐mer peptide and Kelch, with the red dashed line indicating the hydrogen bond. I) ITC experiment of Kelch and E79A mutant, with affinity expressed as the KD value below the curve. J) ITC assays were conducted for Kelch with ponicidin. K) The KD value was determined by Biacore X100 using SPR technique. Different color curves represent different ponicidin concentrations. L) ITC assay was conducted for Δ54‐PGAM5 with ponicidin. M) Ponicidin promoted resistance of Keap1 to different temperature gradients (CETSA). N) Co‐immunoprecipitation assay. O) EMSA experiment of Kelch, Δ54‐PGAM5, and ponicidin. P) Ubiquitination assay. Q) Ponicidin affects Keap1‐PGAM5 protein co‐localization in HepG2 cells.

To better understand the molecular mechanism of the Keap1 and PGAM5 interaction, we conducted structural biology experiments. By sequence alignment, we found that the ESGE motif is conserved across five different species and interacts with Keap1 (**Figure** **4**A,B). To assess the binding affinity of the polypeptides based on the ESGE motif, we synthesized four polypeptides and used ITC experiments. The 20‐mer peptide exhibited the smallest KD value of 107 ± 35 nM and the strongest binding affinity (Figure 4C–F). Crystallographic experiments were conducted to obtain the structure of the Keap1‐PGAM5 complex. The crystal structure of Kelch and an IE 12‐mer peptide was obtained, with a Kelch molecule and an IE 12‐mer peptide found in the asymmetric unit (PDB code: 8IN0). The complex structure had a space group of P61 and only nine residues in the IE 12‐mer peptide could be determined due to stable conformation during binding (Figure 4G). The structure of Kelch consists of a six‐blade propeller and the IE 12‐mer peptide forms a stable hairpin structure when incorporated into the Kelch structure. Specific residues in the IE 12‐mer peptide, such as Val73, Arg74, Lys75, Arg76, and Val78, interact with specific residues on Kelch, including Arg483 and Tyr525, Ser555, Gln530, Tyr334, and Ser602, respectively. Glu79, which is located closest to Kelch's binding cavity, interacts with Asn414 and Arg380 on Kelch (Figure 4H). It is speculated that Glu79 may play a crucial role in the binding of the ESGE motif with Keap1. To further confirm this hypothesis, an E79A mutant based on the 20‐mer peptide was synthesized and ITC experiments were conducted. The results showed that the binding affinity of the E79A mutant was significantly reduced, with a K_D_ value of 37.66 µM, which is a difference from the nM range to the µM range (Figure 4I). These experiments demonstrated that Glu79 is the key residue in Kelch binding to PGAM5. In order to further study the specific targets of ponicidin, we purified the Kelch domain of Keap1 and the ∆54‐PGAM5 (amino acids from number 54 to 289) protein in vitro to explore their binding capacity in the presence of ponicidin. The KD value of Kelch and ponicidin was determined to be 55.8 µM (Figure 4J), We further verified the binding of Kelch and ponicidin by SPR technique, and the affinity constant KD value of the two was 34.98 µM (Figure 4K), while ∆54‐PGAM5 was not found to bind ponicidin (Figure 4L). The CETSA assay revealed an increase in the remaining amount of Keap1 upon the addition of ponicidin (Figure 4M). At the same time, co‐immunoprecipitation was used to verify the interaction between Keap1 and PGAM5, which showed an increase in Keap1 interacting with PGAM5 after the addition of ponicidin (Figure 4N). EMSA results showed that ponicidin could bind to Kelch and promotes its binding with PGAM5 (Figure 4O). Considering that Keap1 is an E3 ligase, it can transfer ubiquitin to the lysine residue of the protein interacting with Keap1, leading to ubiquitination and degradation of the protein. Therefore, we investigated whether Keap1 regulates the ubiquitination of PGAM5. The results showed that ponicidin could increase the ubiquitination of PGAM5 (Figure 4P). In HepG2 cells, we observed a reduction in the protein fluorescence intensity of PGAM5 following ponicidin administration, accompanied by an increase in Keap1‐PGAM5 protein co‐localization (Figure 4Q). These data demonstrate that ponicidin can bind to Keap1, thereby facilitating the interaction between Keap1 and PGAM5 to promote the ubiquitination of PGAM5.

### 2.5 Ponicidin Stabilizes the Binding of Keap1 to PGAM5 by Affecting the Allostery of the Kelch Domain of Keap1

**Figure 5:**
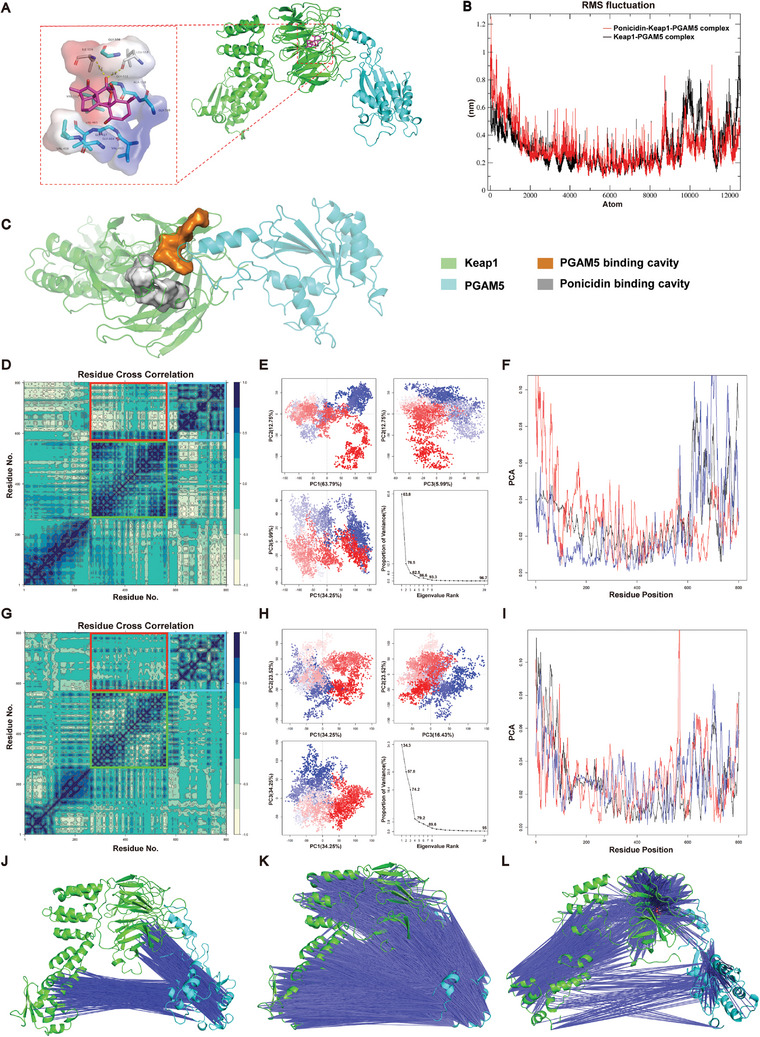
Ponicidin stabilizes the binding of Keap1 to PGAM5 by affecting the allostery of the Kelch domain of Keap1. A) Conformation of last frame in 50 ns molecular dynamics simulation. Red represents negative electrostatic potential energy; blue represents positive electrostatic potential energy. B) RMSF curve of Ponicidin‐Keap1‐PGAM5 complex (red) and Keap1‐PGAM5 complex (black). Atoms from 1 to 8799 are located on Keap1 and atoms from 8800 to 12559 are located on PGAM5. RMSF represents root mean square fluctuation. C) Comparison of the binding cavity of PGAM5 and Keap1 with the binding cavity of Keap1 and ponicidin. D) DCCM analysis matrix of Keap1‐PGAM5 complex, the region of black blue meant the residue pair in horizontal and vertical coordinate have positive correlation in movement patterns, while region of yellow means the negative correlation, the red box represents the overall motion correlation between Kelch and PGAM5, the green box represents the motion correlation of amino acid residues in the Kelch domain, and the blue box represents the motion correlation of amino acid residues in PGAM5. E) PCA analysis of the Keap1‐PGAM5 complex trajectory, the conformation points were colored from blue to red in order of time. F) Contribution of each residue to the first three principal components of Keap1‐PGAM5 complex, black/blue/red lines meant first/second/third principal component. G) DCCM analysis matrix of Ponicidin‐Keap1‐PGAM5 complex, the red box represents the overall motion correlation between Kelch and PGAM5, the green box represents the motion correlation of amino acid residues in the Kelch domain, and the blue box represents the motion correlation of amino acid residues in PGAM5. H) PCA analysis of the Ponicidin‐Keap1‐PGAM5 complex. I) Contribution of each residue to the first three principal components of Ponicidin‐Keap1‐PGAM5 complex. J–L) Movement correlation of each residue with a negative correlation coefficient ranged from −0.8 to −1.0 of Keap1‐PGAM5 complex J), ranged from −0.6 to −0.8 of Keap1‐PGAM5 complex (K), and ranged from −0.6 to −0.8 of Ponicidin‐Keap1‐PGAM5 complex (L). Since the necessary format conversion when using Bio3D, all figures visualized by Bio3D reordered the residues, the first residue existed in protein complex was calculated as residue 1, so residue 1 in these figures made by Bio3D were equal to residue 57 in Keap1, and residue 569 were equal to residue 57 in PGAM5 in other analysis.

To investigate the impact of ponicidin on the interaction between Keap1 and PGAM5, attempts were made to obtain the cocrystal of ponicidin and Keap1‐PGAM5 complex. However, this was unsuccessful. Autodock Vina was used to conduct molecular docking simulations, revealing that ponicidin can stably bind with the Kelch domain of Keap1 (**Figure** **5**A) with a binding affinity of ≈−9.7 kcal mol^−1^ Ponicidin forms hydrogen bonds with Ile559 and Leu557, and interacts hydrophobically with various residues including Gly558, Val512, Gly511, Ala510, Gly509, Val418, Val465, Gly417, Gly464, and Val463. These results suggest that ponicidin selectively binds to Keap1 rather than PGAM5, which supports our experimental findings. The RMSF curve indicates that the Keap1‐PGAM5 complex is more stable after binding with ponicidin, suggesting that ponicidin may stabilize the binding between Keap1 and PGAM5. (Figure 5B). Combined with our previously obtained crystal structure data, it is speculated that ponicidin binds to Ile559 and Leu557 on Keap1. Its binding position is close to the binding area of Keap1 and PGAM5 in the spatial position. This causes the Kelch domain of Keap1 to undergo conformational changes, resulting in a closer binding area of Keap1 and PGAM5. This promotes the binding stability of Keap1 and PGAM5 (Figure 5C). Thus, we investigated the concrete conformational stabilities of Keap1‐PGAM5 and Ponicidin‐Keap1‐PGAM5 complex. Our findings indicate that the binding of ponicidin resulted in a significant decrease in positively correlated movement patterns and an increase in negatively correlated movement patterns in the six‐bladed β‐sheets formed by the Keap1‐Kelch domain. There was a significant decrease in negatively correlated movement patterns between PGAM5 and Keap1. Additionally, the positively correlated movement patterns in residues of PGAM5 were also reduced (Figure 5D–G), changes in the motion patterns of the complex may significantly affect the biological functions of the protein complex. These phenomena demonstrated that the binding of ponicidin influenced the allosteric pattern changes of Kelch domain, and the unstable movement of PGAM5 was inhibited. Specifically, PyMOL visualization showed changes in the negative correlation between residue movements (Figure 5J–L). Principal component analysis (PCA) was used to analyze the conformational changes, and we found that much more principal components could only explain the same movement proportion of variance after the binding of ponicidin (Figure 5E–H). Notably, the first two principal components mainly represented the conformational changes of PGAM5 before the binding of ponicidin. However, this phenomenon was not obvious after the binding of ponicidin (Figure 5F–I). All of these analyses support that ponicidin can affect the allosteric structure of the Kelch domain and promote the binding stability of Keap1 and PGAM5.

### 2.6 Ponicidin Promotes the Tightening of the Interaction Pocket between Keap1 and PGAM5 and Stabilizes the Formation of Keap1‐PGAM5 Complex

**Figure 6:**
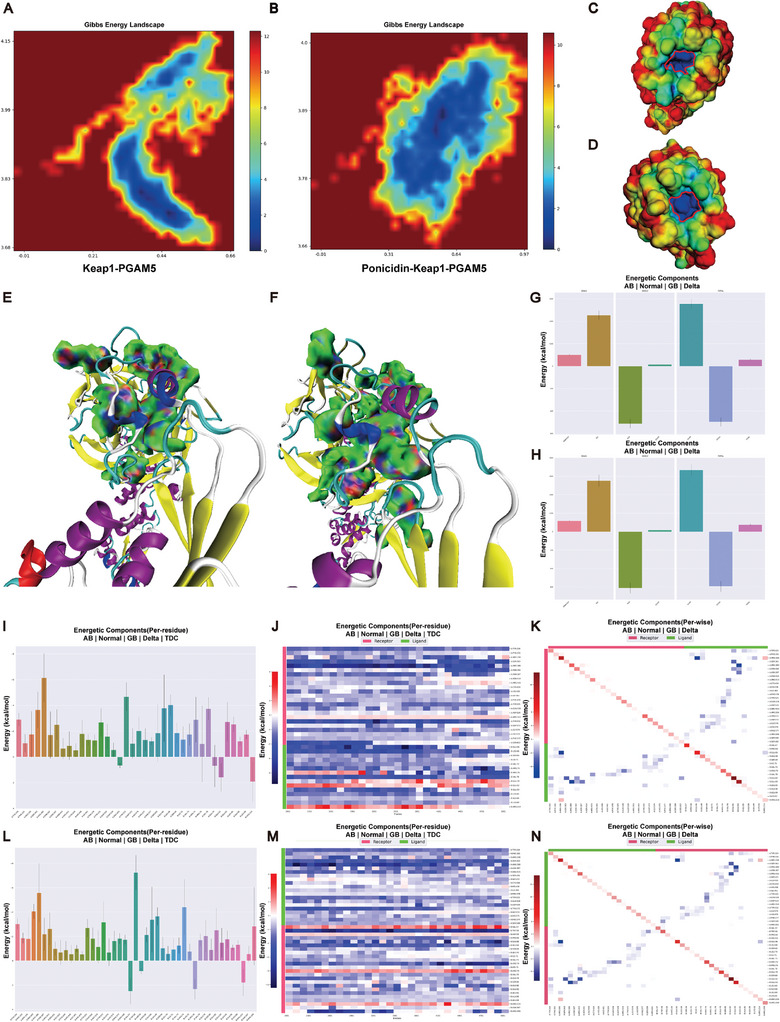
Ponicidin promotes the tightening of the interaction pocket between Keap1 and PGAM5 and stabilizes the formation of Keap1‐PGAM5 complex. A,B) Gibbs free energy landscape of Keap1‐PGAM5 complex (A) and Ponicidin‐Keap1‐PGAM5 complex (B). C,D) Electrostatic potential visualization of Keap1, before (C) and after (D) the binding of ponicidin. The red–green–blue color scheme represents a variation from a negative value to a positive value, and the red cycle was used to highlight region with significantly positive electrostatic potential. E,F) IGM analysis of Keap1‐PGAM5 complex (E) and Ponicidin‐Keap1‐PGAM5 complex (F). The dark Blue region means hydrogen bonds, green region means van der Waals interaction. G,H) Binding energy of Keap1‐PGAM5 complex (G) and Ponicidin‐Keap1‐PGAM5 complex H). VDWAALS means van der Waals interaction, EEL means electrostatic interaction, EGB means polar component of solution energy, ESURF means non‐polar component of solution energy, GGAS = VDWAALS+EEL, GSOLV = EGB+ESURF, TOTAL = GGAS+GSOLV. I–K) Contributions of each residue to binding energy (I), detailed analysis of the binding energy changes during simulation (J), and contribution of residue interactions to binding energy (K) of Keap1‐PGAM5 complex. Chain A = Keap1, Chain B = PGAM5. L–N) Contributions of each residue to binding energy (L), detailed analysis of the binding energy changes during simulation (M), and contribution of residue interactions to binding energy (N) of Ponicidin‐Keap1‐PGAM5 complex. Chain A = Keap1, Chain B = PGAM5.

Based on the studies above, it can be concluded that ponicidin promotes the formation of the Keap1‐PGAM5 complex. To further investigate changes in the interaction pockets of Keap1‐PGAM5 after binding ponicidin, we analyzed the Gibbs free energy landscapes. Our findings indicate that the Keap1‐PGAM5 complex has a more intense energy well after the binding of ponicidin (**Figure** **6**A,B), the disappearance of shallower potential wells suggests that binding with small molecules leads to a more focused conformational distribution of PGAM5. This suggests that the binding of ponicidin alters the conformation distribution of the Keap1‐PGAM5 complex. Ponicidin increased the polarity of the Keap1 binding interface, suggesting that it up‐regulated the electrostatic complementary characteristics (Figure 6C,D). Independent Gradient Model (IGM) analysis was used to visualize non‐bonded interactions. We found that the interaction region, which contains van der Waals interactions and hydrogen bonds, was expanded after the binding of ponicidin (Figure 6E,F). MM/GBSA methodology was used to calculate the binding energy between Keap1 and PGAM5. The Keap1‐PGAM5 binding energy was initially −61.48 kcal mol^−1^, which increased to −75.21 kcal mol^−1^ after ponicidin binding. This increase was due to a significant reduction in electrostatic interaction energy (from −460.96 to −551.72 kcal mol^−1^) and van der Waals interaction energy (from −107.01 to −116.87 kcal mol^−1^) (Figure 6G,H). Free energy decomposition analysis revealed an increase in the residues involved in the Keap1‐PGAM5 interaction (Figure 6I–N). Specifically, we found that the number of residues in PGAM5 that participated in Keap1‐PGAM5 interaction significantly increased. Residues including Trp58, Pro60, Leu70, Lys75, Ser80, Asn267, and Arg269 in PGAM5 formed new interactions in interface after the binding of ponicidin (Figure 6I‐L), and it was obvious that Trp58 provided high and stable binding energy (Figure 6M). These results suggest that the binding of ponicidin strengthened the binding between Keap1 and PGAM5.

### 2.7 Ponicidin Promoted Mitochondrial Apoptosis through the PGAM5‐Related Pathway

**Figure 7:**
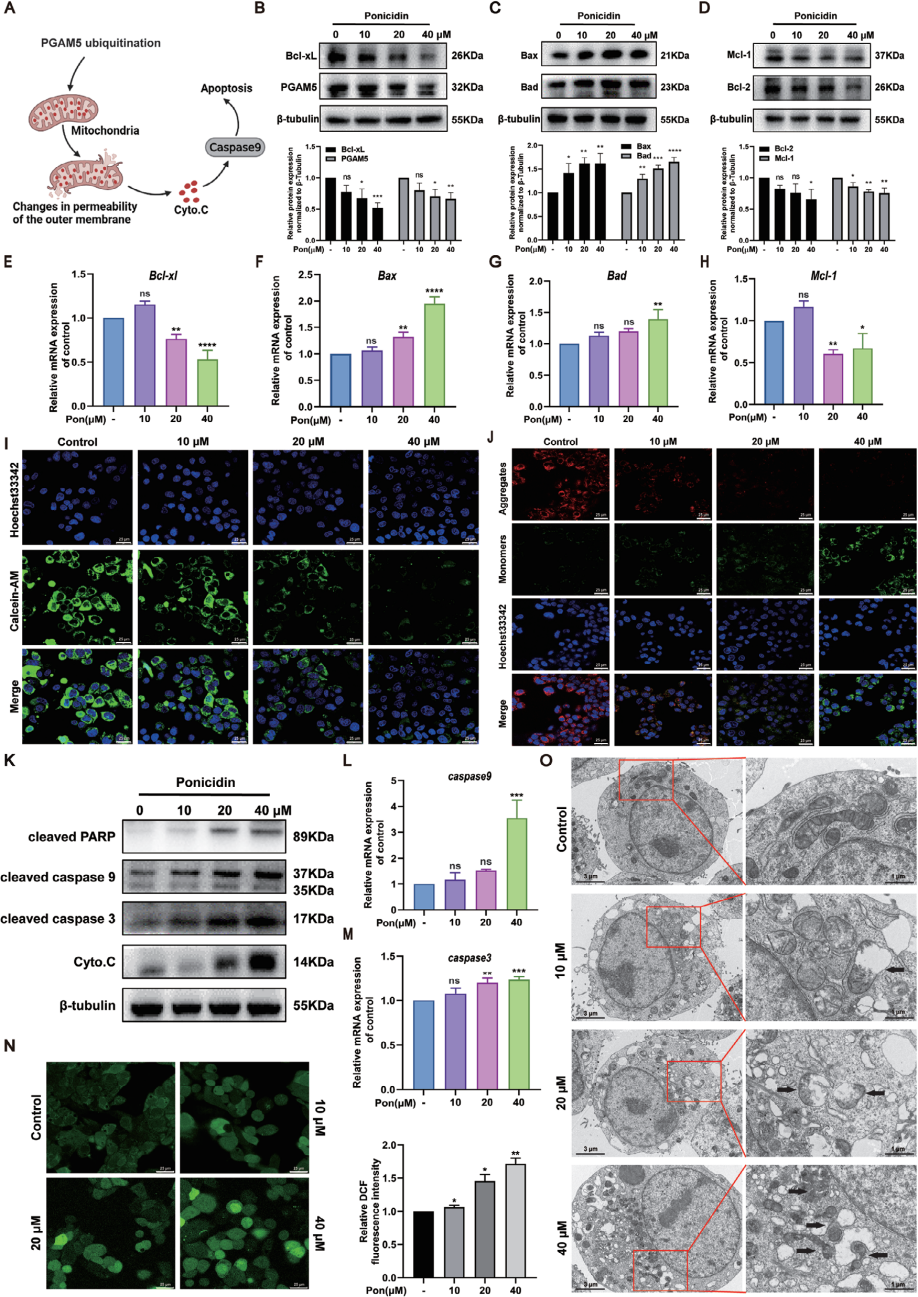
The effect of ponicidin on mitochondrial apoptosis and ROS in HCC cells. A) Schematic diagram of cell apoptosis. B) Protein levels of PGAM5 and Bcl‐xL were evaluated by western blotting (left) and quantified by statistical analysis (right). C) Protein levels of Bad and Bax in HepG2 cells were measured by western blotting (left) and quantified by statistical analysis (right). D) Protein levels of Bcl‐2 and Mcl‐1 were measured by western blotting (left) and quantified by statistical analysis (right). E–H) mRNA levels of *Bcl‐xl*, *Bax*, *Bad* and *Mcl‐1* in HepG2 cells were measured by qPCR, normalized to *GAPDH*. I) Calcein AM fluorescence intensity in HepG2 cells treated with or without ponicidin for 24 h. J) JC‐1 kit used to measure mitochondrial membrane potential in HepG2 cells treated with ponicidin. K) Western blotting to detect protein expression of cleaved PARP, caspase9, caspase3, and Cyto.C in HepG2 cells treated with different doses of ponicidin. L,M) qPCR analysis of *caspase 9* and *caspase 3* mRNA levels. N) DCFH‐DA fluorescent probe used to detect ROS in HepG2 cells treated with ponicidin. O) The effects of ponicidin treatment on the mitochondria of HepG2 cells were investigated using TEM. Black arrows show the location of changes in mitochondrial morphology. These experiments were repeated three times (n = 3), with ^*^P < 0.05, ^**^P < 0.01, ^***^P < 0.001, ^****^P < 0.0001 via one‐way analysis of variance (ANOVA).

PGAM5 can participate in apoptosis by interacting with Bcl‐xL.^[^ ^30^ ^]^ The Bcl‐2 protein family, which functions as an apoptosis regulator, consists of both proapoptotic and prosurvival members. As a result of the Bcl‐2 family driving mitochondrial outer membrane permeabilization, apoptosis‐inducing factor‐dependent and caspase‐dependent apoptotic cell death are activated.^[^ ^31^, ^32^, ^33^ ^]^ Changes in the permeability of the mitochondrial outer membrane result in the release of cytochrome c (Cyto. C) during apoptosis, leading to caspase activation (**Figure** **7**A).^[^ ^34^ ^]^ Previous studies have shown that ponicidin can promote the ubiquitination of PGAM5. Therefore, we speculate that ponicidin may play a role in apoptosis through the ubiquitination of PGAM5. The protein expression levels of apoptosis‐related proteins in the Bcl‐2 family, such as Bax, Bad, Bcl‐2, and Mcl‐1, were detected. Additionally, the expression levels of PGAM5 and Bcl‐xL in HepG2 cells treated with ponicidin were quantified using western blotting. The results showed that the levels of both PGAM5 and Bcl‐xL were significantly lower in the ponicidin‐treated HepG2 cells (0.6‐fold and 0.4‐fold, respectively) (Figure 7B). Treatment with ponicidin resulted in an increase in Bax and Bad expression, and a decrease in Bcl‐2 and Mcl‐1 expression (Figure 7C,D). The same as the level of protein expression, ponicidin significantly decreased *Bcl‐xL* mRNA expression in HepG2 cells (Figure 7E), while the mRNA levels of *Bax* and *Bad* were significantly increased (Figure 7F,G). Furthermore, mRNA expression of *Mcl‐1* was significantly decreased after ponicidin treatment (Figure 7H). These data suggest that ponicidin can trigger apoptosis in HepG2 cells through the modulation of apoptotic proteins in the Bcl‐2 family.

Therefore, we further examined the effect of ponicidin on mitochondria. Initially, we assessed the effect of ponicidin treatment on the opening of the mitochondrial permeability conversion pore (mPTP) in HepG2 cells. The results showed that ponicidin treatment led to a significant reduction in Calcein AM fluorescence intensity compared to the control group, indicating a faster rate of mPTP opening (Figure 7I). The impact of ponicidin on the caspase‐dependent pathway was further evaluated through protein and transcriptional levels of Cyto. C, cleaved caspase 9, caspase 3, and PARP. Ponicidin‐treated HepG2 cells showed significantly higher levels of Cyto. C, cleaved caspase 9, caspase 3, and PARP compared to control cells (Figure 7K). The qPCR data analysis also revealed that ponicidin increased the mRNA levels of *caspase 9* and *caspase 3* in HepG2 cells (Figure 7L,M). The effects of ponicidin treatment on mitochondrial membrane potential and ROS production were assessed. Immunofluorescence experiments revealed a decrease in mitochondrial membrane potential and mitochondrial damage, as evidenced by the decrease in red fluorescence and increase in green fluorescence (Figure 7J). Furthermore, DCFH‐DA fluorescent probe experiments showed an increase in ROS production in HepG2 cells following ponicidin treatment (Figure 7N). The impact of ponicidin treatment on the mitochondria of HepG2 cells was investigated through transmission electron microscopy (TEM) (Figure 7O). The mitochondria in the control group were observed to be normal and neatly arranged, exhibiting clear membranes and typical vacuoles. In contrast, the mitochondrial cristae membranes were reduced and disorganized or even absent following ponicidin treatment. The inner and outer mitochondrial membranes were destroyed, the mitochondria were swollen and ruptured, and larger vesicles were observed concurrently. Collectively, the results indicate that ponicidin treatment activated the caspase‐dependent mitochondrial pathway, leading to mitochondrial damage and ROS production in HepG2 cells.

### 2.8 Ponicidin can Promote HCC Cell Apoptosis and Inhibit Tumor Growth In Vivo

**Figure 8:**
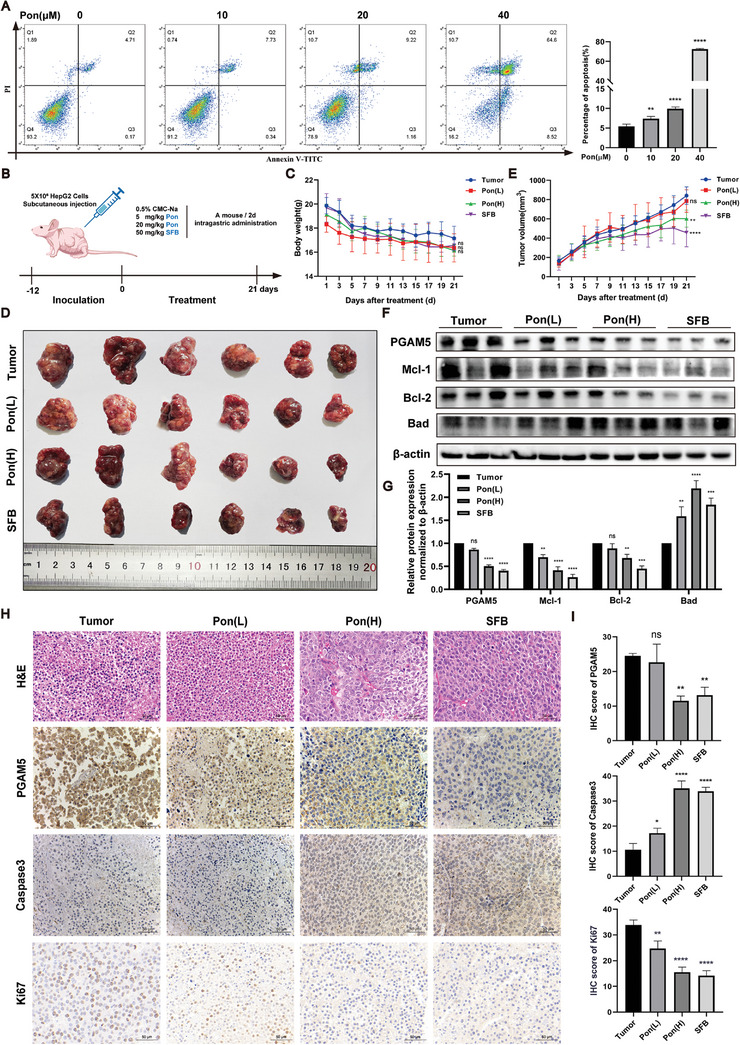
Ponicidin promotes the apoptosis of hepatocellular carcinoma cells by inhibiting the expression of PGAM5 protein and down‐regulating the anti‐apoptotic signaling pathway. A) The influence of ponicidin on the apoptosis of HepG2 cells was evaluated using flow cytometry. B) Experimental procedure of mouse xenograft tumor. C) Weight statistics of mice. D) Tumor map of mice. E) Statistics of tumor growth curve in mice. F) Western blot of PGAM5, Mcl‐1, Bcl‐2, and Bad in the anti‐apoptotic signaling pathway showed a significant decrease in the expression of oridonin. G) Statistical analysis of PGAM5, Mcl‐1, BCL‐2, and Bad protein expression. H) H&E staining and IHC staining of tumor tissues in mice. I) IHC staining statistics of Ki67, PGAM5, and Caspase3 in mouse tumor tissues. These experiments were repeated three times (n = 6), with ^*^P < 0.05, ^**^P < 0.01, ^***^P < 0.001, ^****^P < 0.0001 via one‐way analysis of variance (ANOVA).

The aforementioned studies have demonstrated that ponicidin leads to mitochondrial damage. Consequently, we conducted an in vitro test to investigate whether ponicidin induces apoptosis in HepG2 cells. The results of the flow cytometry assay, which was used to detect apoptosis, indicated that the rate of apoptosis in HepG2 cells increased gradually as the concentration of ponicidin administered increased (**Figure** **8**A). To verify the inhibitory effect of ponicidin on liver cancer cell proliferation, we conducted xenograft experiments on BALB/c nude mice (Figure 8B). We found that different doses of ponicidin did not cause significant changes in the body weight or organ ratios of the mice, indicating that ponicidin has no obvious side effects (Figure 8C; Figure S4A–E, Supporting Information). Meanwhile, it was observed that the tumor volume was significantly reduced after treatment with ponicidin compared to the tumor group, indicating its potential to inhibit tumor growth in vivo (Figure 8D,E). We have demonstrated in cells that ponicidin can promote PGAM5 ubiquitination and activate apoptosis. Therefore, we detected the protein expression levels of PGAM5, Mcl‐1, Bcl‐2, and Bad in tumors, and found that ponicidin could significantly reduce the protein expression levels of PGAM5, Mcl‐1 and Bcl‐2, while increasing the protein expression level of Bad, indicating that ponicidin promoted the apoptosis of HepG2 cells (Figure 8F,G). The H&E staining results indicate that the tumor group had larger nuclei, less cytoplasm, and larger nucleoplasm. Treatment with ponicidin resulted in dense and concentrated nuclear chromatin in tumor tissue, along with nuclear division and eosinophils, suggesting that ponicidin may inhibit tumor growth by inducing tumor cell apoptosis. The IHC results further demonstrate that PGAM5 is highly expressed in HCC tissues, and ponicidin can reduce its protein levels. The results showed that after ponicidin treatment, caspase3 protein levels increased while Ki67 expression decreased (Figure 8H,I). These findings suggest that ponicidin can reduce PGAM5 levels, induce apoptosis, and inhibit the growth and proliferation of HepG2 cells.

## 3 Discussion

Our investigative studies have demonstrated the efficacy of ponicidin in the treatment of a range of malignant tumors, including pancreatic cancer cell growth,^[^ ^24^ ^]^ gastric cancer,^[^ ^25^ ^]^ colon cancer cells,^[^ ^18^, ^26^ ^]^ gallbladder cancer,^[^ ^35^ ^]^ and human breast cancer.^[^ ^36^ ^]^ However, its role and mechanism in HCC remain unclear. In this study, we first assayed ponicidin and found that it could inhibit the growth and proliferation of HCC cells. By using biotin to label ponicidin, we discovered that ponicidin could bind to Keap1 in HepG2 cells using target fishing technology. However, our analysis revealed that the correlation between Keap1 expression and survival time in HCC patients was not statistically significant. Consequently, we conducted further research and analyzed the mass spectrometry results, which indicated that PGAM5 can bind to Keap1 and can be pulled down by ponicidin in HepG2 cell lysate. PGAM5 has been studied for its role in various cellular processes on the mitochondrial membrane, particularly in liver damage.^[^ ^37^, ^38^, ^39^ ^]^ However, the role of PGAM5 in cancer has not been extensively researched. In this study, we found that PGAM5 expression is elevated in HCC tissues and that high expression of PGAM5 is correlated with poor survival in HCC patients. Utilizing the HuProt™ Human Proteome Microarray, we discovered that ponicidin exhibited binding affinity for Keap1, but not for PGAM5. This result differs from that obtained by target fishing, which demonstrated the ability to pull down both Keap1 and PGAM5. Therefore, it can be postulated that ponicidin may bind to Keap1 and influence the interactions between Keap1 and PGAM5. These results suggest that PGAM5 could serve as a predictive biomarker and therapeutic target in HCC patients.

Apoptosis can be divided into endogenous apoptotic pathways mediated by mitochondria and exogenous apoptotic pathways mediated by death receptors. The endogenous apoptotic pathway mainly involves the release of Bcl‐2 family from mitochondria, such as Bcl‐2, Bcl‐xL and Bax, which interact with each other to increase mitochondrial outer membrane permeability and release Cyto. C, which aggregates the precursor of caspase 9 and activates it through self‐catalysis, subsequently activating effector protein caspase 3.^[^ ^29^ ^]^ The exogenous apoptotic pathway recruits Fas associated protein with a novel death domain through the binding of death receptors and ligands, inducing the activation of caspase 8. Subsequently, the activated caspase 8 cleaves activates caspase‐3, thereby executing the exogenous apoptotic pathway.^[^ ^40^ ^]^ It has been demonstrated that ponicidin induces apoptosis by inhibiting the expression of Bcl‐2 and Survivin,^[^ ^41^ ^]^ and that Survivin regulates apoptosis by binding to and blocking the initiator caspase 9 and effector caspase 3.^[^ ^42^ ^]^ In our study, we found that ponicidin promotes apoptosis in HCC cells by stabilizing the formation of the Keap1‐PGAM5 complex and activating the caspase‐dependent mitochondrial apoptosis pathway. We also observed that ponicidin promoted the ubiquitination of PGAM5 and decreased the expression of PGAM5. Our experiments suggested that ponicidin targets Keap1, and structural and dynamic simulations further support the notion that ponicidin promotes the formation of the Keap1‐PGAM5 complex. Consequently, the efficacy of the drug is contingent upon its capacity to influence the formation of the complex, thereby establishing a novel mechanism of drug action.

To elucidate the molecular mechanism of the Keap1 and PGAM5 interaction, we employed molecular dynamics simulations and AlphaFold3 to predict the Keap1 and PGAM5 interaction. Based on these findings, we proceeded to synthesize the PGAM5 peptide sequence and resolve the complex crystal structure of the Kelch structural domain of Keap1 and the PGAM5 peptide for the first time. The resolved crystal structure indicates that it contains a six‐bladed propeller, formed by the Keap1‐Kelch structural domain and the PGAM5 polypeptide. The ESGE motif of PGMA5 is similar to the ETGE motif of Nrf2.^[^ ^43^ ^]^ In the crystal structure of the peptides derived from the ETGE motifs containing the Keap1‐Kelch structural domain and Nrf2, the side chain of Glu 79 in the ETGE is deeply buried in the pocket. Among them, the Glu79 of PGAM5 mainly interacts with the Arg380 and Asn414 of Kelch by hydrogen bonding, whereas the Glu79 of Nrf2 mainly interacts with the R415, R483, and Ser555 of Kelch by hydrogen bonding,^[^ ^44^ ^]^ which interacts with distinct amino acids, as well as suggests that the binding pockets where the Glu79 is located are inconsistent. It was also found that the Glu79 mutant greatly weakened the binding affinity, suggesting that Glu79 was a key residue involved in their interaction. This finding is consistent with previous research in this area.^[^ ^45^ ^]^ Meanwhile, we used molecular dynamics simulation to understand the specific binding mode of ponicidin and Keap1‐PGAM5 complex. Our results further demonstrate that ponicidin can stabilize the Keap1‐PGAM5 complex without direct interaction with PGAM5. Ponicidin may bind to the Kelch domain of Keap1, causing the conformational change of the Kelch domain, resulting in a tighter binding pocket of Keap1 and PGAM5, and enhancing the binding stability of Keap1 and PGAM5. Our experimental results are supported by molecular dynamics simulations, which provide further insight into the binding mode between Keap1 and PGAM5.

In summary, this study provides insight into the pharmacological activities of ponicidin and the Keap1‐PGAM5 complex. Ponicidin promotes the interaction of Keap1 and PGAM5, leading to the activation of the mitochondrial apoptosis pathway. Residue Glu79 on PGAM5 plays a critical role in complex formation. these findings have implications for the development of new therapeutic strategies for HCC and other cancers.

## 4 Experimental Section

### Reagents and Antibodies

Ponicidin (B0296) was obtained from Must Biotechnology (Chengdu, China). The antibodies for Keap1 (10503‐2‐AP, Proteintech, Wuhan, China), PGAM5 (28445‐1‐AP, Proteintech, Wuhan, China), Artificially synthesized PGAM5 peptide (Nanjing Yuanpeptide Biotech Co., LTD), Bcl‐xL (T40057, Abmart, Shanghai, China), Bcl‐2 (T40056, Abmart, Shanghai, China), Mcl‐1 (T40058, Abmart, Shanghai, China), Bax (T40051, Abmart, Shanghai, China), Bad (T40052, Abmart, Shanghai, China), caspase 9 (T40046, Abmart, Shanghai, China), caspase 3 (T40044, Abmart, Shanghai, China), cytochrome c (T55734, Abmart, Shanghai, China), Ki67(GB111499, Servicebio, Wuhan, China), β‐tubulin (M30109, Abmart, Shanghai, China), MMP9 (3852, CST, Massachusetts, MA, USA), P21 (sc‐6246, Santa Cruz, California, USA) and PCNA (sc‐6246, Santa Cruz, California, USA) were used for western blotting.

### Animal and Drug Administration

The protocol for the animal experiment was approved by the Animal Ethics Use Committee of the International Institute for Translational Chinese Medicine, Guangzhou University of Chinese Medicine. BALB/c nude wild‐type male specific pathogen‐free (SPF) mice aged 4–6 weeks were provided by Guangdong Medical Laboratory Animal Center, License No.: SYXK (Guangdong) 2019‐0144. Animal ethics approval number: 20230615. BALB /c nude mice were injected with a suitable number of HepG2 cells in logarithmic growth phase into the armpits of BALB/c nude mice, and tumors were formed 12 days later. The successfully modeled mice were divided into 4 groups: 1) Tumor group (n = 6), 0.5% CMC‐Na solution (i.g) was given every 2 days; 2) Ponicidin low‐dose group (n = 6): 5 mg kg^−1^ Ponicidin solution (i.g) was given every 2 days; 3) Ponicidin high‐dose group (n = 6): 20 mg kg^−1^ ponicidin solution (i.g) was given every 2 days; (4) Positive drug group (n = 6): 70 mg kg^−1^ SFB solution (i.g) was given every 2 days. Ponicidin and SFB were dissolved in 0.5% CMC‐Na solution.

### Cell Culture

American Type Culture Collection (ATCC) provided human liver cancer cell lines HepG2, MHCC97H, MHCC97L, and normal hepatocyte WRL‐68 cells. A DMEM medium (G4517, Servicebio, Wuhan, China) supplemented with 10% fetal bovine serum (12483020, Gibco, New York, USA) and 1% penicillin‐streptomycin (P1400, Solarbio, Beijing, China) was used for cell culture. The cells were incubated in a humidified incubator containing 5% CO₂ at 37 °C. The medium was replaced every two days, and the cells were passaged using trypsin‐EDTA (Gibco) at ≈80% confluence. All reagents utilized in the cell culture process were procured from commercial sources and underwent sterile filtration prior to use.

### Cell Viability Measurement

After seeding the cells in 96‐well plates, the cells were incubated overnight and treated with ponicidin at various concentrations for 24 h. Following this, the MTT assay was performed according to the manufacturer's protocol to determine the percentage of viable cells compared to the control group.

### Western Blotting Analysis

Following the administration of ponicidin to HepG2 cells for a period of 24 h, the cells were lysed using a RIPA lysate. The lysates were then centrifuged, and the protein concentration was determined and quantified using Thomas Brilliant Blue G250. The protein lysates were then separated by SDS‐PAGE and transferred to PVDF membranes. After sealing the PVDF membrane with 5% skimmed milk, the membrane was incubated with the primary antibody (diluted 1:1000) overnight at 4 °C. The membrane was then incubated with the secondary antibody (diluted 1:5000) for 2 h at room temperature. The detection of the protein bands was performed using an ECL chemiluminescence system, and the protein levels were determined using Image J software. β‐tubulin was employed as a control for upsampling.

### EdU Assay

Cell proliferation was determined via EdU incorporation assay (C0071S, Beyotime, Shanghai, China). The 4 × 10^3^ cells were incubated with ponicidin for 24 h, and then treated with 10 µM EdU for 2 h. After a 15‐min fixation with 4% paraformaldehyde at room temperature. The cells were permeabilized by 0.3% Triton X‐100 for 15 min, and then incubated with 50 µL of click reaction solution for 30 min. The cell nuclei were counterstained with Hoechst 33342 for 10 min. Edu was detected via inverted fluorescent microscopy.

### Transwell Migration Assay

Transwell chambers were procured from Corning, New York, USA. Prior to the commencement of the experiment, HepG2 cells were subjected to an 8 h starvation period in serum‐free media. This was followed by the addition of ponicidin to the upper chamber of the Transwell apparatus. A total of 200 µL of media were added to the upper transwell chamber, containing ≈1×10^5^ HepG2 cells. A total of 600 µL of medium containing 10% FBS was added to the bottom chamber (24‐well) for incubation. The incubation was conducted at 37 °C and 5% CO_2_ for 48 h. To quantify cell migration, the wells containing HepG2 cells were fixed with 4% paraformaldehyde and stained with crystal violet. The crystal violet surface area was extrapolated from 10x zoom microscopy images.

### The synthesis of Biotin Labeled Ponicidin (Bio‐Ponicidin)

Ponicidin (23.0 mg, 0.063 mmol, 1eq), biotin‐PEG4‐acid (34.3 mg, 0.070 mmol, 1.1eq) and DCC(16.2 mg, 0.079 mmol, 1.25eq) was dissolved in the dry DCM solution. The coupling reaction was carried out at 60 °C for 48 h under the catalysis of 0.1eq DMAP. After rotating evaporation of DCM solvent, the crude product was purified by reversed‐phase silica gel column chromatography using the mixture of MeOH/H_2_O (3:1) to give a white solid as Bio‐Ponicidin (26.7 mg). The structure of Bio‐Ponicidin was verified by ^1^H‐NMR and high‐resolution mass spectrometry. ^1^H NMR (400 MHz, Chloroform‐d) δ 6.98 (t, *J* = 5.5 Hz, 1H), 6.21 (s, 1H), 5.23 (s, 1H), 4.51 (dd, *J* = 7.8, 4.9 Hz, 1H), 4.41 – 4.31 (m, 1H), 4.16 – 4.03 (m, 1H), 3.83 (t, *J* = 5.9 Hz, 2H), 3.64 (d, *J* = 1.9 Hz, 13H), 3.57 (t, *J* = 5.0 Hz, 2H), 3.45 (p, *J* = 5.0 Hz, 2H), 3.16 (td, *J* = 7.4, 4.5 Hz, 1H), 2.92 (dd, *J* = 12.8, 4.9 Hz, 1H), 2.75 (d, *J* = 12.8 Hz, 1H), 2.69 (t, *J* = 5.9 Hz, 2H), 2.26 (t, *J* = 7.4 Hz, 2H), 1.85 – 1.60 (m, 14H), 1.46 (p, *J* = 7.5 Hz, 2H), 1.40 – 1.06 (m, 10H). HRMS (ESI) m/z: [M+H]^+^ Calcd for C_41_H_61_N_3_O_13_SH^+^ 836.3998; Found 836.3984.

### Target Fishing

Lysis of HepG2 cells using RIPA buffer. Bio‐Ponicidin was added to the cell lysates and incubated for 1.5 h, followed by the addition of streptavidin magnetic beads and another 1.5 h of incubation. Unbound proteins were removed using a magnetic separator and PBS was used to wash the streptavidin magnetic beads three times. Proteins bound to ponicidin were identified using silver staining and then analyzed by mass spectrometry by Oebiotech (Shanghai, China).

### Tissue Microarray and Immunohistochemistry

Tissue microarrays (TMAs) were obtained from Shanghai Outdo Biotech Company and approved by their Ethics Committee of Shanghai Outdo Biotech Company (Approval No. SHXC2021YF01). The liver cancer tissue samples for TMA were prepared by dewaxing and antigen retrieval prior to immunostaining. PGAM5 antibody (Proteintech, 1:5000 dilution) and Keap1 antibody (Proteintech, 1:3000 dilution) were used to detect the protein in the liver cancer tissue samples. Microscopy images were captured using an upright microscope system (Nikon, Japan). The stained slides were analyzed using Image J software to determine the percentage of positive staining in each sample. The TMA results were evaluated by a pathologist who was blinded to the clinical data, with a cut‐off value of 20% defining high and low expression of PGAM5.

### Ponicidin Detection of HuProt^TM^ Human Proteome Microarray

The HuProt^TM^ Human Proteome Microarray V4.0 (Guangzhou GeneCloud Bio. Co., Ltd., China) was employed to identify proteins that interact with ponicidin. The microarrays were blocked with blocking buffer for 3 h, after which they were incubated with 10 µM Bio‐Ponicidin or free biotin for 1 h. Following washing, the microarrays were incubated. Subsequently, the microarrays were incubated with Cy3‐streptavidin (Cy3‐SA) incubation solution at room temperature for 1 h. Finally, the microarrays were incubated with a Molecular Devices GenePix 4000B scanner (Axon Instruments, Sunnyvale, CA) to identify ponicidin‐interacting proteins. The data were acquired by GenePix Pro v6.0 software. In order to eliminate the inhomogeneous signal between different protein spots within the same chip due to inconsistent background values, the background correction method was employed. This was achieved by calculating the ratio of the foreground value to the background value for each protein, designated as F/B. Based on this, the SNR was defined as the average value of F/B for two replicate protein spots. For any protein, the cutoff threshold was set by taking the SNR value of the sample protein as the calculation, and defining the sample (Bio‐Ponicidin) cutoff = mean + 2sd (95% CI) = 13, that is, a sample (Bio‐Ponicidin) SNR ≥ 13 is a potential positive protein of the sample.

### Computational Simulations of Complex

To predict the structure and study the dynamics of Keap1 and PGAM5, we employed a combination of the Alphafold database, Gromacs 2020.4 software, and ZDOCK Server v3.0.2. The Alphafold database was used to predict the structures of Keap1 and PGAM5, which were then refined with energy minimization using Gromacs 2020.4 and the AMBER99SB force field. Protein‐protein docking of Keap1 and PGAM5 was performed using ZDOCK Server v3.0.2, Arg380 in Keap1, and Glu79 in PGAM5 were selected as binding site residues, based on our experimental results. The structure was selected with the best docking score for molecular dynamics simulation with Gromacs 2020.4, including energy minimization, NVT and NPT equilibration, and 50 ns of formal molecular dynamics. To analyze the simulation results, we RMSD, Radius of gyration (Rg), hydrogen bonds curves, clusters of motion mode, and Gibbs energy landscape using Gromacs 2020.4. The complex structures of various clusters and those with the lowest Gibbs energy were visualized using PyMOL 2.4.0 software. Finally, LigPlot software was used to create a 2D diagram that clarified the concrete interactions in the interface of the Keap1‐PGAM5 complex with the lowest Gibbs energy.

### Expression and Purification of Protein

Genes encoding the Keap1‐Kelch (Gene ID: 9817, amino acid residues from number 309 to 624) and PGAM5 domain (Gene ID: 192111, amino acid residues from number 54 to 289) were cloned into a pET‐28a expression vector (Novagen, Germany), and then transformed into *Escherichia coli* strain BL21 (Codonplus) cells. The cells were then incubated at 18 °C for 12 h with 0.1 mM isopropyl‐β‐thiogalactoside (IPTG) to induce protein expression. The proteins were purified using Ni‐NTA affinity resin (Qiagen, Germany), while proteins were further purified using HiTrap QHP 1 mL (GE, USA) and Superdex 200 Increase 10/300GL resin (GE, USA). Protein purity was determined by 12% SDS‐PAGE.

### Cocrystal Structure of Kelch and PGAM5‐Derived Peptide

For crystal formation, Kelch was incubated with the PGAM5 IE 12‐mer peptide on ice for 30 min and crystallized using the sitting drop steam diffusion method. The crystals of the Kelch and IE 12‐mer peptide complex were grown at room temperature in a solution containing 0.8 µL of ligand‐protein and a 0.8 µL reservoir consisting of 0.95 M lithium sulfate, 0.5 M ammonium sulfate, and 0.1 M sodium citrate at pH 5.4. The crystals reached diffraction mass after 3 days.

### Data Collection and Structure Determination

X‐ray diffraction data was collected using an Xcalibur Nova diffractometer (Oxford Diffraction) and the Kelch protein structure (PDB code: 7ECA) was utilized as the initial search template for molecular replacement via the MOLREP function in the CCP4 software. The structures were manually refined and fitted using CCP4 and Coot, respectively. The final structure of the Kelch and IE‐12mer peptide complex in the asymmetric unit consisted of 2304 protein atoms, 20 ligand molecules, and 314 water molecules. The R_work_ and R_free_ values were 0.2490 and 0.2852, respectively. The statistical data of structural refinement are provided in **Table** **1**.

**Table 1:** Data collection and refinement statistics. (PDB code: 8IN0).

Crystals	Kelch with 12‐mer Peptide
**Data collection**	
Space group	P6_1_
a, b, c (Å)	103.655 103.655 56.1673
α, β, γ (°)	90.00, 90.00, 120.00
Resolution (Å)	24.69‐1.8 (1.864‐1.8)^a)^
R_merge_ ^b)^	0.1477 (1.136)
I/σ_(I)_	14.68 (1.49)
Completeness (%)	97.14 (99.91)
Redundancy	9.2 (5.8)
	**Refinement**
Resolution (Å)	24.69‐1.8
No. reflections	31129 (3193)
R_work_ ^c)^/R_free_ ^d)^	0.2490/0.2852
No. atoms	2638
Protein	2304
Ligand	20
Water molecules	314
**B‐factors** (**Å ^2^**)	
Protein	18.55
Ligand	35.84
Water molecules	30.73
Bond lengths (Å)	0.014
Bond angles (°)	1.91
Ramachandran favored (%)	95.25
allowed	4.41
Outliers (%)	0.34

^a)^ Values in parentheses are for the highest‐resolution shell;

^b)^ R_merge_ = Σ |(I – < I >)|/σ(I), where I is the observed intensity;

^c)^ R_work_ = Σ_hkl_ ||Fo| – |Fc||/ Σ_hkl_ |Fo|, calculated from working data set;

^d^ R_free_ is calculated from 5.0% of data randomly chosen and not included in refinement.

### Isothermal Titration Calorimetry (ITC)

The Kelch proteins and peptides were diluted in Tris‐HCl buffer (20 mm Tris‐HCl and 150 mm NaCl), and ITC experiments were performed using The MicroCal ITC200 (Malvern, UK). The sample pool was injected with 300 µL of soluble Kelch via droplets at 2‐min intervals for 19 times. ITC was conducted at 25 °C with a reference power of 9.98 µcal s^−1^. K_D_ values were obtained via MicroCal analysis software, and GraphPad Prism 9.0 was used for image processing.

### Simulations of Binding Mode between Keap1‐PGAM5 Complex

The molecular docking of ponicidin and Keap1‐PGAM5 was performed using Autodock Vina 1.1.2 software, the centroid of grid box was selected in Keap1 pocket. The grid was defined with 40 points in each of the x, y, and z dimensions. The spacing between these grid points was set to 0.375 Å. This configuration results in a 3D grid used to determine the search space for molecular docking simulations. and the complex with the best docking score was selected for molecular dynamics simulation using Gromacs 2020.4 software with the AMBER99SB force field. The molecular dynamics simulation was run for 50 ns, and Gromacs 2020.4 was used to calculate RMSD, Rg, RMSF, and hydrogen bond curves. The complex structure was visualized using PyMOL 4.6.0 software.

### Surface Plasmon Resonance Assay (SPR)

The SPR experiments were conducted using Biacore X100 (GE Healthcare, Boston, MA, USA). The optimal pH of Kelch protein was determined to be 4.5 by pre‐concentration of the protein. The Kelch protein was then immobilized on a CM5 chip through an amine coupling kit. Ponicidin was diluted in PBS with 0.5% DMSO at different concentrations. The chip surface was flowed with various concentrations of ponicidin at a constant rate, and the instrument recorded the corresponding response value at steady state. The Biacore X100 analysis software was used to calculate the affinity constant K_D_ value, while GraphPad Prism 9.0 was used for image processing.

### Cellular Thermal Shift Assay

HepG2 cell lysate was mixed evenly with 10 µm ponicidin and incubated for 30 min. An equal volume of DMSO was added to the blank group. Seven temperature gradients (40, 50, 60, 70, 80, 90, and 100 °C) were set in the gradient PCR instrument. The samples were heated at the set temperature position for 15 min, stabilized at 25 °C for 3 min, and stored at 4 °C. After centrifugation at 14000 rpm, the supernatant was prepared for western blot analyst using a Keap1 antibody.

### Co‐Immuniciprecipitation Assay

HepG2 cells were treated with ponicidin for 24 h and lysed with NP‐40 buffer. After ultrasonic and centrifugation, the supernatants were incubated with Keap1 antibody and protein A/G agarose beads overnight at 4 °C. The beads were washed with NP‐40 buffer and boiled with SDS loading buffer for 10 min at 95 °C prior to western blotting.

### Electrophoretic Mobility Shift Assay (EMSA)

The protein was incubated with the ponicidin on ice for 30 min, followed by the addition of 5 x loading buffer without SDS and through mixing. The sample was separated by electrophoresis on a 7% non‐denatured gel at 4 °C and 90 V for 1 h. The gel was stained with Coomassie brilliant blue staining solution, followed by decolorization and analysis using a gel imaging system (BIO‐RAD, USA).

### Ubiquitination Assay

HepG2 cells were treated with ponicidin for 24 h and lysed with 400 µl NP‐40. After ultrasonic and centrifugation, the supernatants were incubated with a PGAM5 antibody and protein A/G agarose bead overnight at 4 °C. The beads were washed with NP‐40 buffer and boiled with SDS loading buffer for 10 min at 95 °C prior to western blotting.

### Immunofluorescence Co‐Localization Analysis

HepG2 cells were treated with ponicidin for 24 h. Following a 30‐min fixation period with 4% paraformaldehyde, the cells were permeabilized with 1% Triton X‐100 The cells were blocked with 5% BSA for 30 min, followed by an overnight incubation with Keap1 and PGAM5 antibodies. The cells were then incubated with fluorescent antibodies for 2 h. Finally, the nuclei were stained with DAPI. A laser confocal fluorescence microscope (Leica TCS SP8, Germany) was employed to photograph and analyze the co‐localization of Keap1 and PGAM5.

### Simulations of Binding Mode between Ponicidin‐Keap1‐PGAM5 Complex

R package Bio3D (http://thegrantlab.org/bio3d_v2/tutorials/) was used to draw a dynamical cross‐correlation matrix (DCCM) and to perform PCA analysis of MD trajectories of Keap1‐PGAM5 and Ponicidin‐Keap1‐PGAM5 complex. The residues’ dynamic correlations were visualized by PyMOL to clarify the flexibility of each complex. The concrete contribution of each residue to each principal component was also visualized by Bio3D.

### Calculated Electrostatic Potential by Solving Poisson–Boltzmann Equation

Gibbs energy landscape was calculated by Gromacs v2020.6 software. The electrostatic potential energy surface was calculated by APBS v3.4.1 platform, which calculated electrostatic potential by solving Poisson–Boltzmann equation (https://server.poissonboltzmann.org/apbs). In visualization, the minimum surface potential was selected as −2 kT/e, and the maximum surface potential was selected as 2 kT/e. Independent Gradient Model (IGM) analysis was finished by Multiwfn v 3.8(dev) software (http://sobereva.com/multiwfn/), using promolecular density. The visualization of IGM analysis was finished by VMD v1.9.3 software (https://www.ks.uiuc.edu/Research/vmd/), the drawing method was selected as “Isosurface”, and the Isovalue was equal to 0.001. MM/GBSA methodology in gmx_MMPBSA v1.6.2 software (https://valdes‐tresanco‐ms.github.io/gmx_MMPBSA/dev/) was used to calculated the binding energy of Keap1 and PGAM5, and GB‐OBC2 model was used.

### Quantitative Polymerase Chain Reaction

RNA was extracted from HepG2 cells using the AG RNAex Pro Reagent (AG21102, Accurate Biology, Changsha, China), and the concentration was quantified using a nucleic acid meter. Reverse transcription of cDNA was conducted in accordance with the instructions provided in the manufacturer's protocol for the Evo M‐MLV reverse transcription kit (AG11705, Accurate Biology, Changsha, China). The CT values were obtained, and the relative mRNA expression was calculated using the 2‐ΔΔCT method. The primer sequences utilized in the experiment are presented in **Table** **2**.

**Table 2:** Sequence (5′‐3′) of primers used for real time quantitative PCR.

Gene	Forward (5′ → 3′)	Reverse (5′ → 3′)
GAPDH	TGTGGGCATCAATGGATTTGG	ACACCATGTATTCCGGGTCAAT
Keap1	GTGGCGAATGATCACAGCAA	GGACGTAGATTCTCCCCTGG
PGAM5	CTGTCTCTGATCAACGTGCG	GTGGTACTGGGAATGCCTGA
Bcl‐xL	AGCTTGGATGGCCACTTACCTG	TGCTGCATTGTTCCCATAGAGTTC
Mcl‐1	ATCCATGTTTTCAGCGACGG	TGCTAATGGTTCGATGCAGC
Bax	AACATGGAGCTGCAGAGGAT	CCAATGTCCAGCCCATGATG
Bad	CGGAGGATGAGTGACGAGTT	AAGTTCCGATCCCACCAGG
Caspase 9	ACTAACAGGCAAGCAGCAAAG	TCACCAAATCCTCCAGAACCA
Caspase 3	GTAGAGAACTGTGGAGTGAT	TGCATTCGCTTGTAGGATACT

### Mitochondrial Permeability Transition Pore

To measure mitochondrial permeability transition pore (mPTP), an mPTP Assay Kit (C2009S, Beyotime, Shanghai, China) was used. Cells were incubated with Calcein AM staining solution and a fluorescent quenching working solution of CoCl_2_ in the dark for 45 min. The staining solution was then replaced with fresh DMEM medium and incubated in the dark for a further 30 min at 37 °C. The cells were then counterstained with Hoechst 33342 for 15 min. Calcein AM and Hoechst 33342 fluorescence were captured on a confocal microscope.

### Measurement of Mitochondrial Membrane Potential

To assess the mitochondrial membrane potential, a commercially available kit, the JC‐1 Mitochondrial Membrane Potential Assay Kit (C2006, Beyotime, Shanghai, China), was utilized. The JC‐1 dye was applied to HepG2 cells for a period of 30 min in an environment maintained at 37 °C in the dark. Subsequently, the supernatant was aspirated, and the cells were washed twice with PBS. Subsequently, the cells were counterstained with Hoechst 33342 for 20 min, followed by two washes with PBS. The JC‐1 dye aggregates and monomer were visualized using laser confocal microscopy.

### ROS Detection

The ROS Detection Kit (S0033S, Beijing, China) was utilized to identify ROS in accordance with the provided guidelines. The cells were initially plated in a small dish and subsequently treated with ponicidin for a period of 24 h. Thereafter, the cells were incubated with 10 µm DCFH‐DA at 37 °C for a duration of 20 min. The levels of ROS production were quantified using a laser confocal fluorescence microscope (Carl Zeiss, Jena, Germany), with the fluorescence intensity of DCF serving as the metric for evaluating ROS production.

### Transmission Electron Microscopy (TEM)

Following the treatment of HepG2 cells with varying concentrations of ponicidin, de‐medium was added to 2.5% electron microscopy fixative (G1102, Servicebio) and re‐suspended and mixed for fixation at 4 °C for a period of 2–4 h. Pre‐embedding was then carried out using 1% agar for post‐fixation. Dehydration was achieved through the use of alcohol of varying concentrations at room temperature. Polymerization was conducted in an oven at 60 °C following embedding with 812 embedding agent (90529‐77‐4, SPI). Subsequently, the sections were sectioned and stained. Finally, the samples were observed under a transmission electron microscope (HT7800/HT7700, Hitachi) and images were captured for analysis.

### Flow Cytometry

The HepG2 cells were treated with the indicated concentrations of ponicidin for a period of 24 h. To assess cell apoptosis, the cells were stained using the Annexin V‐FITC/PI Apoptosis Detection Kit (MA0220, Meilune, China) and analyzed using the BD Accuri Flow Cytometer (BD Accuri C5, USA).

### Hematoxylin‐Eosin (H&E) Staining

The tumor tissue samples from nude mice were fixed in 4% paraformaldehyde, followed by embedding in paraffin, freezing sectioning, and staining with H&E.

### Statistics Analysis

Data were analyzed via GraphPad Prism 9.0 software and are presented as mean ± SD. Independent sample t‐test or one‐way analysis of variance (ANOVA) were as necessary, with *p* < 0.05 being statistically significant.

## Conflict of Interest

The authors declare no conflict of interest.

## Author Contributions

B.Z., Z.L., and L.Z. are co‐first authors and contributed equally to this work. B.X.Z., Z.H.L. and L.S.Z. performed the experiments; R.Z., C.Y.W., and Z.Q.L. designed the project and revised the manuscript; B.X.Z., Z.H.L., L.S.Z., L.J., Y.H.X., and Y.Z, performed the informatics analysis and experiments; B.X.Z. and C.Y.W. wrote the paper. All authors read and approved the final manuscript.

## Supporting information

Supporting Information

## Data Availability

The data that support the findings of this study are available from the corresponding author upon reasonable request.
