# Supplementary material for: Ponicidin Promotes Hepatocellular Carcinoma Mitochondrial Apoptosis by Stabilizing Keap1‐PGAM5 Complex
Source: Adv Sci (Weinh). 2024 Aug 8;11(38):2406080. doi: 10.1002/advs.202406080 (PMC11481384; doi:10.1002/advs.202406080)
Supplement: Supplementary file 1 — Supporting Information [file ADVS-11-2406080-s001.docx]

**Supporting Information**

**Ponicidin promotes hepatocellular carcinoma mitochondrial apoptosis by stabilizing Keap1-PGAM5 complex**

*Bixin Zhao^1#^, Zuhui Liang^1#^, Lisheng Zhang^2#^, Lin Jiang^1^, Yuanhang Xu^1^, Ying Zhang^1^, Rong Zhang^1*^, Caiyan Wang^1*^ and Zhongqiu Liu^1*^*

^1^ State Key Laboratory of Traditional Chinese Medicine Syndrome, International Institute for Translational Chinese Medicine, Guangzhou University of Chinese Medicine, Guangzhou 510006, China.

^2^ Research Center of Integrative Medicine, School of Basic Medical Science, Guangzhou University of Chinese Medicine, Guangzhou 510006, China.


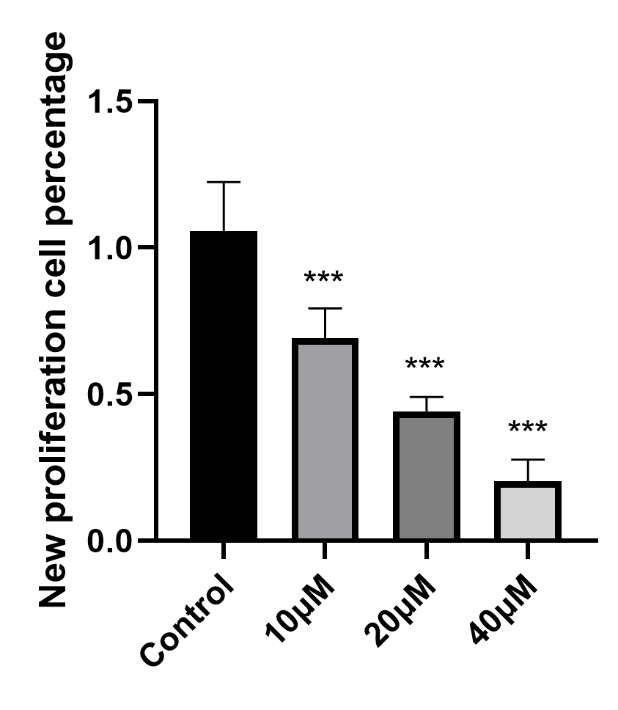


**Figure S1**: Statistical analysis of the percentage of newly proliferating cells in EdU experiments. These experiments were repeated three times (n=3), with *^*^P* < 0.05, ^*^*^*^P* < 0.01, *^***^P* < 0.001 via one-way analysis of variance (ANOVA).

**Figure S2**: Differential positive proteins (SNP ≥ 13) bound to Bio-ponicidin were detected using human proteomic microarrays. Red triangles indicate Keap1 proteins.PGAM5 with SNP=2.2,is not shown in the figure.

**
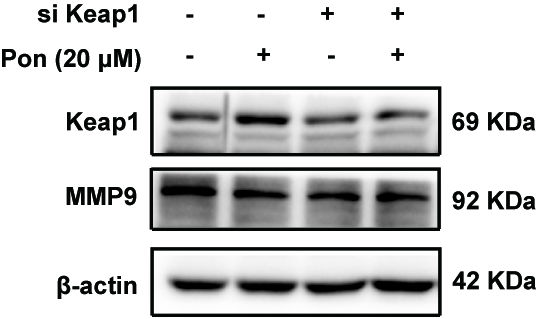
**

**Figure S3** The impact of ponicidin on MMP9 protein expression in HepG2 cells was evaluated following si Keap1. Treatment of HepG2 cells with ponicidin resulted in a significant reduction in MMP9 protein expression. However, following si Keap1, ponicidin treatment exhibited no discernible effect on MMP9 protein expression.


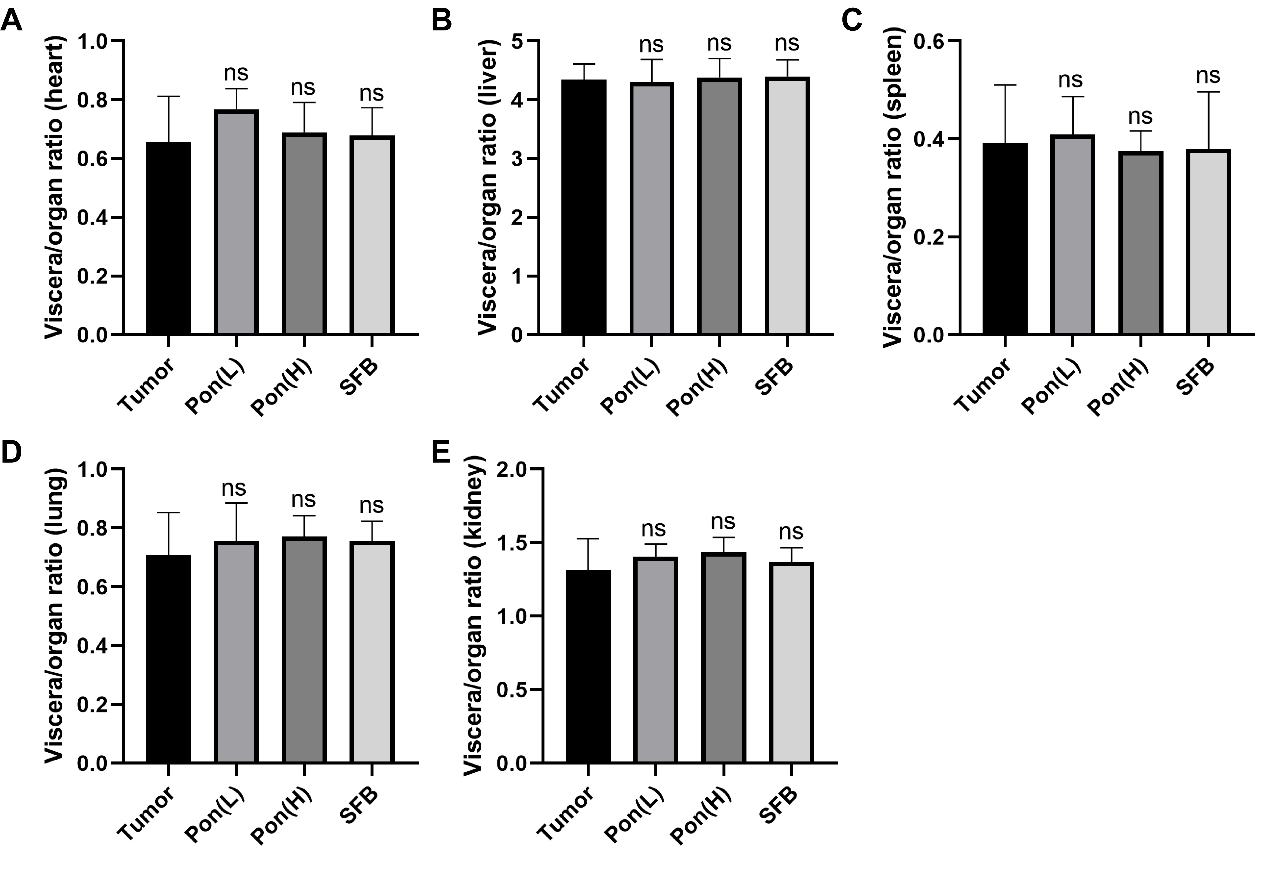


**Figure S4**: Mouse organ ratios after treatment with different drugs. (A) Heart-to-weight ratio. (B) Liver weight ratio. (C) Spleen weight ratio. (D) Lung weight ratio. (E) Kidney weight ratio. These experiments were repeated three times (n=6), with with *^*^P* < 0.05, ^*^*^*^P* < 0.01, *^***^P* < 0.001 via one-way analysis of variance (ANOVA).
